# Strategien und Effekte digitaler Interventionen bei der Übergewichts- und Adipositastherapie von Kindern und Jugendlichen – ein systematischer Review

**DOI:** 10.1007/s00103-022-03512-3

**Published:** 2022-03-23

**Authors:** Sabine Pawellek, Alexandra Ziegeldorf, Hagen Wulff

**Affiliations:** 1 Institut für Gesundheitssport und Public Health, Universität Leipzig, Jahnallee 59, 04109 Leipzig, Deutschland; 2 Gesundheitserziehung/Gesundheitsbildung, Universität Potsdam, Potsdam, Deutschland

**Keywords:** Gewichtsreduktion, Kindes- und Jugendalter, Medien, Lebensstilintervention, Body-Mass-Index, Weight loss, Childhood and adolescence, Media, Lifestyle intervention, Body mass index

## Abstract

**Hintergrund:**

Steigende Adipositasprävalenzen im Kindes- und Jugendalter sind geprägt von ungesunden Lebensweisen wie geringer Bewegung durch hohen Medienkonsum. Neueste Studien nutzen die Erreichbarkeit dieser Zielgruppe durch digitale Medien, womit Technologien neue Ansätze in der Interventionsgestaltung der Gewichtsreduktion darstellen. Allerdings stellt sich die Frage, welche digitalen Kombinationen und methodischen Programmkonzepte effektive Body-Mass-Index(BMI)-Veränderungen bedingen.

**Ziel:**

Um Erkenntnisse über effektive Maßnahmengestaltung und Medieneinsatz zu gewinnen, sollen digitale Interventionsstrategien zur BMI-Reduktion übergewichtiger Kinder und Jugendlicher analysiert und bewertet werden.

**Methoden:**

Ein systematischer Review wurde in den Datenbanken Medline via PubMed, Science Direct und Web of Science zur Analyse von Studien aus den Jahren 2016 bis 2021 über Veränderungen im BMI und BMI-Z-Score von übergewichtigen und adipösen 6‑ bis 18-Jährigen durchgeführt. Die methodische Studienqualität wurde nach den Richtlinien des Cochrane Risk of Bias bewertet.

**Ergebnisse:**

Aus 3974 Studien wurden 7 Artikel identifiziert, die den Einsatz von Fitnessarmbändern, Smartphones und computerbasierten Programmen beschreiben. Alle Medien erzielten BMI-Reduktionen, wobei Smartphoneinterventionen via Anrufe und Nachrichten die signifikantesten Veränderungen bewirkten.

**Diskussion:**

Smartphones bieten als Anbieter digitaler Programme (z. B. Apps) effektive Ansatzpunkte zur Adipositasreduktion. Auf Basis der Datenlage bestätigt sich neben der Auswahl und der Kombination mehrerer Medien die Relevanz des Familieneinbezugs und die methodische Fundierung der Maßnahmen. Aufgrund des jungen Alters der Teilnehmenden müssen mediale Interventionen zielgruppengerecht zugänglich gemacht werden.

## Abstract (en)

**Background:**

Rising obesity prevalence in childhood and adolescence are characterized by unhealthy lifestyles such as low physical activity due to high media use. Recent studies use the accessibility of this target group through digital media, whereby technologies represent new approaches in the intervention design of weight reduction. However, the question arises as to which digital combinations and methodological program concepts condition effective body mass index (BMI) changes.

**Aim:**

To gain insights into effective program design and media use; digital intervention strategies for BMI reduction in overweight and obese children and adolescents will be analyzed and evaluated.

**Methods:**

A systematic review was conducted in the databases MEDLINE via PubMed, Science Direct, and Web of Science to analyze studies from 2016 to 2021 on changes in BMI and BMI z‑score of overweight and obese 6‑ to 18-year-olds. Methodological study quality was assessed according to the Cochrane Risk of Bias guidelines.

**Results:**

From 3974 studies, seven articles describing the use of fitness wristbands, smartphones, and computer-based programs were identified. All media achieved BMI reductions, with smartphone interventions via calls and messages causing the most significant changes.

**Discussion:**

Smartphones as providers of digital programs (e.g., apps) offer effective approaches to obesity reduction. Based on the data, the selection and combination of several media as well as the relevance of family involvement and the methodological foundation of the measures are confirmed. Due to the young age of the participants, media interventions must be made accessible to the target group.

## Hintergrund

Adipositas, definiert als pathologisch erhöhtes Körpergewicht bei gegebener Körpergröße, betrifft international über alle Altersgruppen hinweg die Gesamtbevölkerung [1, 2]. Analysen der Fallzahlen bestätigten einen mit steigendem Lebensalter korrelierenden Anstieg der Prävalenz [1]. Vor diesem Hintergrund gewinnt die Betrachtung von Kindern und Jugendlichen an Bedeutung [3]. Adipositas erhöht das Risiko für Folgeerkrankungen wie Diabetes mellitus Typ 2 und Bluthochdruck [4, 5], die das Gesundheitssystem belasten. Die Möglichkeiten einer frühzeitigen Adipositasprävention müssen daher analysiert werden. [6].

### Digitale Medien – Einfluss und Chance

Einflussfaktoren auf die Gewichtsentwicklung lassen sich im individuellen Lebensstil betroffener Kinder und Jugendlichen finden. Dieser Lebensstil ist durch eine aufgrund technischer und kultureller Entwicklungen zunehmende Digitalisierung in den verschiedensten Bereichen des Alltags geprägt [7]. Der digitale Medienkonsum beispielsweise wurde in der Vergangenheit als ein Hauptfaktor für Bewegungsmangel identifiziert, da die Nutzung digitaler Geräte wie Fernseher, Smartphones oder Spielkonsolen die Notwendigkeit körperlicher Aktivität einschränken [8].

Neuere Studien zeigen jedoch kontroverse Ergebnisse. Sie bestätigen einen Bewegungsanstieg durch die Nutzung digitaler Interventionen, womit Medien als Chance statt Risiko für die Gewichtsreduktion fungieren [9, 10]. Neben Vorteilen für Verhaltensveränderungen bergen Medien zusätzlich Potenziale zum Ausgleich individueller Verhältnisse. Strukturell schwach erschlossene Gebiete weisen Probleme auf, Gesundheitsversorgung für Betroffene zugänglich zu machen [11]. Im Kindes- und Jugendalter limitiert fehlende Mobilität somit Partizipationschancen hinsichtlich der Nutzung von Interventionen [12]. Vor dem Hintergrund der Überwindung infrastruktureller Barrieren bestätigen Studien den Einsatz von Technologien als Interventionsinstrument. Die damit gesteigerte Zugänglichkeit zur Zielgruppe birgt Potenzial für eine Stärkung von Programmteilnahme und -effizienz [13].

### Ziele des systematischen Reviews

In der Adipositasforschung stellt sich die Frage, wo Medien ihren Platz in Body-Mass-Index(BMI)-reduzierenden Programmen finden. Systematische Reviews generierten bereits Evidenzen im Erwachsenenalter [14]. Im Kindes- und Jugendalter hingegen bestehen lediglich Übersichtsarbeiten über analoge Behandlungsmöglichkeiten der Adipositas, welche positive Effekte der diät- und bewegungsbezogenen Maßnahmen unterstreichen [15, 16]. Diese konnten im Einklang mit bestehenden Leitlinien der Deutschen Adipositas-Gesellschaft (DAG) erste Anhaltspunkte bezüglich der Relevanz von Einflussfaktoren, wie der Einbezug der Familie, identifizieren [15, 17]. Allerdings fehlt eine Übersicht über digitale Adipositasprogramme [18]. Durchgeführte Literaturarbeiten für diese Altersgruppe umschließen technologisch überholte Methoden (zum Beispiel einfache Schrittzähler), welche der digitalen Revolution nicht gerecht werden [19, 20].

Um diese Lücke zu schließen, wurde in der vorliegenden Studie die Effektivität bestehender, digitaler Übergewichts- und Adipositasinterventionen im Kindes- und Jugendalter in Bezug auf eine Verringerung des BMI anhand von Publikationen aus dem Suchzeitraum 2016 bis 2021 untersucht. Um weiterführend Vorschläge zur Optimierung der Adipositasbehandlung zu formulieren, werden aus der Analyse resultierende Erkenntnisse schrittweise folgende Fragen beantworten:

1. Welche Medien werden für Interventionen zur effektiven BMI-Reduktion verwendet?
2. Welche methodische Interventionsgestaltung findet erfolgreich Verwendung?
3. Welche Ableitungen lassen sich aus den Ergebnissen für Forschung und Praxis treffen?

## Methodik

### Auswahl der Studien

Die systematische Übersichtsarbeit wurde in Anlehnung an die deutschen PRISMA(Preferred Reporting Items for Systematic Reviews and Meta-Analysis)-Richtlinien formuliert [21]. Zur Generierung aktueller Daten wurde der Zeitraum nach den Cochrane-Empfehlungen auf Publikationen zwischen 2016 und 2021 begrenzt [22]. Durchsucht wurden die Datenbanken Medline via PubMed, Web of Science und Science Direct. Zusätzlich wurde eine Freitextsuche durchgeführt.

Eingeschlossen wurden Studien mit Teilnehmenden zwischen 6 und 18 Jahren (Tab. 1). Einschlusskriterien wurden auf Basis des US-amerikanischen Referenzsystems gewählt, welches Übergewicht ab einem BMI >25 kg/m^2^ und Z‑Score >1 SD (Standardabweichung) und Adipositas ab einem BMI von >30 kg/m^2^ und Z‑Score >2 SD definiert. Während der BMI Größe und Gewicht ins Verhältnis setzt, identifiziert der Z‑Score die Standardabweichung des BMI von der Referenzpopulation gleichaltriger und -geschlechtlicher Kinder [23]. Gründe für die Wahl des BMI lagen in der objektiven und zeitsparenden Datenerhebung der Parameter. Analysierte Studientypen waren englischsprachige randomisierte kontrollierte Studien (Randomized Controlled Trials, RCT), welche Adipositas- und Übergewichtsinterventionen durchführten. Ausgeschlossen wurden Teilnehmende mit weiteren Erkrankungen.

**Table 1:** 

Auswahlparameter	Einschlusskriterien	Ausschlusskriterien
Partizipierende	Zwischen 6 und 18 Jahren BMI >25 kg/m^2^ oder	Alter <6 Jahre/>18 Jahre
	BMI-Z-Score >1 SD zu Interventionsbeginn	BMI <25 kg/m^2^ oder BMI-Z-Score <1 SD zu Interventionsbeginn
		Erkrankung an anderen Krankheitsbildern
Intervention	Medial gestützt	Ohne Einsatz von Medien/Technologien
Studiendesign	Randomisierte kontrollierte Studien	Metaanalysen, Systematic Reviews
Studienziel	Veränderung des BMI oder BMI-Z-Scores	Veränderung von Verhalten/Lebensstil

*BMI* Body-Mass-Index, *SD* Standardabweichung

Für eine effektive Suchstrategie wurden mithilfe von MeSH-Terms und booleschen Operatoren die folgenden Schlagworte kombiniert: child, adolescent, obesity, therapy, intervention, media, digital technology, digital device, body mass index, randomized controlled trial. Eine transparente Darstellung der Suchmethodik erfolgt in Tab. 2.

**Table 2:** 

Datenbank	Suchstring	Filter	Stichtag
Medline via PubMed	(Child[Mesh]) OR (Child*) OR (Adolescent[Mesh]) OR (Adolescent*) AND (Obesity[Mesh]) OR (Obesit*) AND (therapy[Subheading]) OR (therap*) OR (therapy[Text Word]) AND (digital media[Text Word]) OR (digital media) OR (digital intervention[Text Word]) OR (digital intervention) OR (Digital Technology[Mesh]) OR (digital device*) OR (digital devices[Text Word]) OR (digital Technolog*) AND (BMI[TextWord]) OR (body mass index[TextWord]) OR (Body Mass Index[Mesh]) AND (RCT) OR (randomized controlled trial)	Publication Date: 5 Years	12.08.2021
		Article Type: RCT	
		Language: English	
		Age: Adolescent: 13–18, Child: 6–12	
Science Direct	(Child OR adolescent) AND (obesity) AND (therapy) AND (digital media OR digital intervention OR digital technology) AND (body mass index) AND (randomized controlled trial)	Publication Years: 2016–2021	26.08.2021
		Subject Areas: Medicine and Dentistry, Nursing and Health Professions, Neuroscience, Agricultural and Biological Sciences, Biochemistry Genetics and Molecular Biology, Pharmacology Toxicology and Pharmaceutical Science, Psychology, Engineering, Immunology and Microbiology, Computer Science	
Web of Science	(Child[Mesh]) OR (Child*) OR (Adolescent[Mesh]) OR (Adolescent*) AND (Obesity[Mesh]) OR (Obesit*) AND (therapy[Subheading]) OR (therap*) OR (therapy[Text Word]) AND (digital media[Text Word]) OR (digital media) OR (digital intervention[Text Word]) OR (digital intervention) OR (Digital Technology[Mesh]) OR (digital device*) OR (digital devices[Text Word]) OR (digital Technolog*) AND (BMI[TextWord]) OR (body mass index[TextWord]) OR (Body Mass Index[Mesh]) AND (RCT) OR (randomized controlled trial)	Open Access	28.08.2021
		Publication Years: 2016, 2017, 2018, 2019, 2020, 2021	
		Document Type: Research Article	
		Search Within all fields: RCT	
		Language: English	
Google Scholar	(Child* OR children OR child OR adolescent) AND (obesity OR Obes*) AND (therapy) AND (intervention) AND (digital OR media OR digital intervention OR digital technology) AND (body mass index OR BMI*) AND (randomized controlled trial)	Publication Years: 2016, 2017, 2018, 2019, 2020, 2021	03.09.2021

*BMI* Body-Mass-Index, *RCT* randomisierte kontrollierte Studie

Eine Genehmigung der Ethikkommission musste aufgrund der Nutzung bereits publizierter Studien nicht beantragt werden.

### Datenauswertung und Qualitätssicherung

Zur Sicherstellung der methodologischen Studienqualität wurde eine Bewertung des Risikos systematischer Fehler (Risk of Bias) nach den Richtlinien von Cochrane Deutschland durchgeführt [24]. Beurteilt wurden extrahierte Artikel hinsichtlich der Randomisierung, Verblindung und Vollständigkeit der Daten. Studien wurden mit hohem, niedrigem oder unklarem Risiko beurteilt.

### Datensynthese

Tabellen wurden entwickelt, um Daten der Studien zusammenzufassen und chronologisch nach dem Erscheinungsdatum zu listen. Individuelle Interventionskonzepte wurden hinsichtlich ihres Aufbaus und ihrer Effektivität analysiert. Eingesetzte Medien wurden herausgearbeitet.

## Ergebnisse

**Figure 1:**
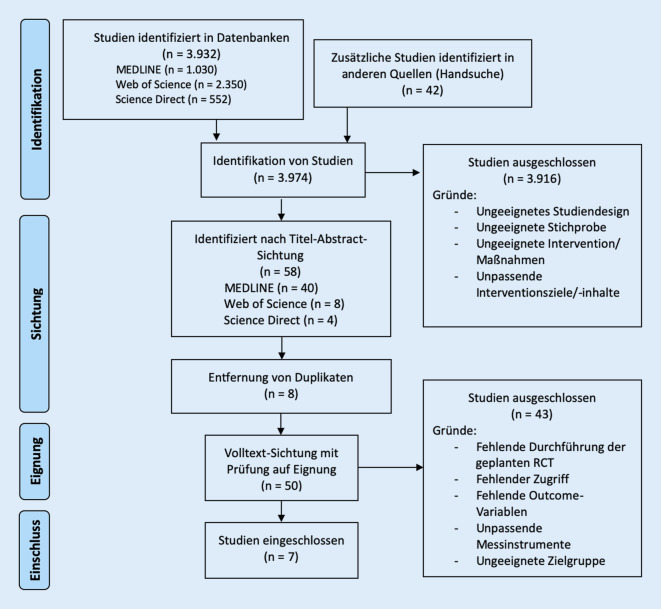

Insgesamt konnten in der Suche 3974 Studien erfasst und gescreent werden. Der Prozess von der Identifikation passender Studien bis zum finalen Einschluss ist in Abb. 1 dargestellt.

### Charakteristika eingeschlossener Studien

Tab. 3 beschreibt die Charakteristika der 7 inkludierten Studien. Die US-amerikanische Population wird in 3 Artikeln analysiert [25–27]. Zudem liegen Interventionsergebnisse aus Griechenland [28], Italien [29], Malaysia [30] und der Türkei [31] vor. Die Länge der Interventionen umfasste zwischen 3 und 12 Monate. Die Studien untersuchten einmal eine 8‑monatige sowie jeweils zweimal eine 3‑, eine 6‑ und eine 12-monatige Zeitperiode.

**Table 3:** 

Studie	Population	Dauer	Verteilung	Verteilung nach Gewicht		Inhalt – IG	Inhalt – KG	Rücklaufquote
Mameli et al., 2016 Italien	10–17 Jahre	3 Monate	*n* = 43, IG = 23, KG = 20	k. D.	k. D.	Armband, App und SMS mit personalisiertem Feedback (1-mal pro Woche)	Empfehlung für Diät und körperliche Aktivität	69,8 %
Fleischman et al., 2017 USA	10–17 Jahre	12 Monate	*n* = 38, IG = 21, KG = 17	k. D.	k. D.	Klinik- und Telebesuch von Adipositasfachpersonal über Vidyo Desktop® (Vidyo, Inc., Hackensack, NJ, USA) und SMS	Klinikbesuche	80 %
Wylie-Rosett et al., 2018 USA	7–12 Jahre	12 Monate	*n* = 366, IG = 184, KG = 182	k. D.	k. D.	Aufklärungsgespräche mit geschultem Fachpersonal; Besprechung partiell über Telefon	Besuche und Gespräch für Eltern und Kindern	> 80 %
Ahmad et al., 2018 *Malaysia*	8–11 Jahre	8 Monate	*n* = 122, IG = 64, KG = 58	ÜG = 41,8 % AP = 58,2 %	ÜG = 40,3 % AP = 59,7 %	50 % persönliche/50 % Facebook-Schulungen und Diskussion über WhatsApp (WhatsApp Inc./Meta Platforms, CA, USA)-Gruppe der Eltern	Warteliste, um Intervention zu späterem Zeitpunkt zu erhalten	91 %
Moschonis et al., 2019 Griechenland	6–12 Jahre	3 Monate	*n* = 80, IG = 40, KG = 40	ÜG = 42,4 % AP = 57,6 %	ÜG = 35,7 % AP = 64,3 %	Computerbasiertes Programm (DST) zur Gestaltung persönlicher Lebensstilempfehlung/Diätoptimierung	Generelle Empfehlung zu einem gesunden Lebensstil	81 %
Chen et al., 2019 USA	13–18 Jahre	6 Monate	*n* = 40, IG = 23, KG = 17	k. D.	k. D.	Fitbit Flex (Fitbit Inc, CA, USA) für 6 Monate, 8 Onlinelernmodule und Erinnerungs-SMS	Pedometer und Essens‑/Aktivitätstagebuch	90 %
Köse et al., 2020 Türkei	12–18 Jahre	6 Monate	*n* = 80, IG = 43, KG = 37	k. D.	k. D.	Motivationales Interview und Erinnerungsnachrichten via SMS	Keine Unterstützung	80 %

*AP* Adipositas, *IG* Interventionsgruppe, *k. D.* keine Details, *KG* Kontrollgruppe, *n* Anzahl der Teilnehmenden, *SMS* Textnachricht, *ÜG* Übergewicht

### Charakteristika der Studienpopulation

Die Stichprobengröße variierte zwischen 31 und 366 Studienteilnehmenden, wobei die durchschnittliche Probandenanzahl 110 betrug. Das Alter der Studienpopulation betrug zwischen 6 und 18 Jahren (Tab. 3). Am häufigsten wurden 10- bis 12-Jährige in Analysen einbezogen. 2 Studien klassifizierten ihre Studienpopulation nach Gewicht. In Relation zu übergewichtigen Kindern und Jugendlichen stellten adipöse Teilnehmende in der Interventionsgruppe einen Anteil von 58,2 % [30] beziehungsweise 57,6 % [28] dar. Auch in der Kontrollgruppe bildeten Menschen mit Adipositas mit jeweils 59,7 % [30] beziehungsweise 64,3 % [28] die Mehrheit.

Von 761 untersuchten Teilnehmenden der eingeschlossenen Studien erhielten 389 eine digital gestützte Interventionsform. Alle RCT unterteilten ihre Studienpopulation in eine Interventions- und eine Kontrollgruppe. Eingesetzte Medien in der Experimentalgruppe waren digitale Armbänder, Smartphones und Telefone, Computer sowie Tablets. Die Kontrollgruppe erhielt entweder keine Unterstützung, Empfehlungen zu einem gesunden Lebensstil, Tagebücher und Arbeitsblätter zur Dokumentation, regelmäßige Besuche bei Adipositasspezialist:innen ohne digitalen Einfluss oder befand sich auf der Warteliste, um das Programm nach Abschluss der primären Interventionsdauer zu durchlaufen. Angaben zur Programmadhärenz wurden in einer Studie aufgeteilt auf spezifisch genutzte Medien gemacht [30].

### Veränderungen des BMI

Als Veränderungsparameter wurden der BMI (Körpergewicht/Körpergröße^2^; 2 Studien) und der BMI-Z-Score (5) genutzt. Der BMI wurde in 57,1 % der Datenerhebungen signifikant reduziert [26, 27, 30, 31]. Aufgrund der unterschiedlichen Darstellungen der BMI-Werte in inkludierten Studien wurden Ergebnisse entweder mit konkretem BMI zum Interventionsende (Post-BMI; 4) oder mit der absoluten Parameterveränderung (3) abgebildet (Tab. 4). In einer der 7 Studien erreichte die Kontrollgruppe eine höhere BMI-Reduktion als die Interventionsgruppe [29]. 42,9 % der untersuchten Ergebnisse wiesen einen Gruppen×Zeit-Effekt auf [27, 30, 31].

**Table 4:** 

Studie	Parameter	Gruppe	Prä-BMI	Post-BMI	∆BMI	Zeiteffekt	Zeit×*Gruppen-Effekt
Mameli et al., 2016	BMI-Z-Score	IG	2,20 (± 0,47)	k. D.	−0,03	↓	Nein
		KG	2,09 (± 0,34)	k. D.	−0,04	↓	
Fleischman et al., 2017	BMI-Z-Score	IG	2,11 (± 0,07)	k. D.	−0,11 (± 0,05)	↓*	Nein
		KG	2,10 (± 0,07)	k. D.	−0,11 (± 0,05)	↓*	
Wylie-Rosett et al., 2018	BMI-Z-Score	IG	2,02 (± 0,39)	k. D.	−0,15	↓*	Nein
		KG	1,95 (± 0,42)	k. D.	−0,12	↓*	
Ahmad et al., 2018	BMI-Z-Score	IG	2,00 (± 0,40)	1,95 (± 0,45)	k. D.	↓*	Ja*
		KG	2,10 (± 0,39)	2,09 (± 0,35)	k. D.	–	
Moschonis et al., 2019	BMI-Z-Score	IG	2,60 (± 0,20)	2,50 (± 0,10)	k. D.	↓	Nein
		KG	2,80 (± 0,20)	2,80 (± 0,20)	k. D.	–	
Chen et al., 2019	BMI	IG	27,37 (± 3,26)	26,93 (± 3,43)	k. D.	↓	Ja*
		KG	28,35 (± 4,36)	29,18 (± 3,88)	k. D.	↑	
Köse et al., 2020	BMI	IG	29,91 (± 3,42)	27,25 (± 2,79)	k. D.	↓*	Ja*
		KG	30,81 (± 3,41)	30,29 (± 3,65)	k. D.	↓	

*BMI* Body-Mass-Index, ↑ Steigerung, ↓ Verringerung, * signifikant, *IG* Interventionsgruppe, *KG* Kontrollgruppe, *k. D.* keine Details, *Prä‑/Post-BMI* BMI vor/nach Interventionsmaßnahmen, *∆BMI* absolute Differenz zwischen Prä- und Post-BMI

### Darstellung des effektiven Medieneinsatzes

Die Verwendung von Medien zeigte einheitlich eine BMI-Reduktion in der Studienpopulation. Der Großteil der Studien nutzte die Kombination aus verschiedenen Geräten (5 Studien), wobei das Smartphone am häufigsten genutzt wurde (5). Signifikante BMI-Veränderungen erzielte die Versendung von Textnachrichten (SMS) via Smartphone.

Köse und Yıldız [31] führten mit 12- bis 14-Jährigen ein analoges motivationales Interview, in welchem Prioritäten und Hindernisse der individuellen Übergewichtsreduktion thematisiert wurden. In 8 Sitzungen führte spezielles Fachpersonal Befragungen durch mit dem Fokus auf körperliche Aktivität, Ernährung und Stressmanagement. Unterstützend dazu erhielten Teilnehmende über 6 Monate 2‑mal pro Woche SMS, welche an Aktivitätssteigerung und gesunde Lebensweisen erinnerten.

Eine andere erfolgreiche Smartphonenutzung wurde mit Telebesuchen gestaltet. Fleischman et al. [26] teilten die Studienpopulation altersspezifisch in 2 Interventions- und Kontrollgruppen. Die erste Gruppe umfasste jeweils 10- bis 13-Jährige, die zweite 14- bis 17-Jährige. Quartalsgebunden besuchten Teilnehmende analog eine Klinik, in welcher Fortschritte in der Gewichtsreduktion evaluiert wurden. Interventionsgruppen erhielten zusätzlich Telebesuche und -beratungen mit Übergewichtsexpert:innen während der 6‑monatigen Interventionszeit. In diesen wurde die Limitierung von Lebensmitteln mit hohem glykämischen Index empfohlen, zudem wurde für eine Verhaltensveränderung auf eine Erhöhung körperlicher Aktivität hingewiesen.

Auch Wylie-Rosett et al. [27] erzielten mit Telefonberatungen signifikante Gewichtsreduktionen. Während Teilnehmende quartalsmäßig durchgeführte analoge Standardbetreuungen durchliefen, erhielt die Interventionsgruppe 8 zusätzliche Beratungen, von welchen die Hauptsitzungen telemedizinisch durchgeführt wurden. Diese thematisierten die Motivation zur Verhaltensveränderung für Familien und beinhalteten auch separate Aufklärungsgespräche für Eltern über ihre Verantwortung für das Gewichtsmanagement ihrer Kinder.

Soziale Medien wurden im Rahmen der Analyse als signifikantes Interventionsinstrument zur Übergewichtsreduktion identifiziert [30]. Die hier untersuchte Studienpopulation wurde zunächst 2‑mal wöchentlich über Steigerung von körperlicher Aktivität und Reduktion sitzender Tätigkeiten aufgeklärt. Dies fand im Wechsel persönlich und online über die Plattform Facebook statt. Zudem wurde eine WhatsApp-Gruppe für Eltern eingerichtet, in welcher wöchentlich gesundheitsbezogene Informationen erschienen und durch Fachpersonal zur Diskussion aufgerufen wurde.

### Nachweis der Studienqualität

**Figure 2:**
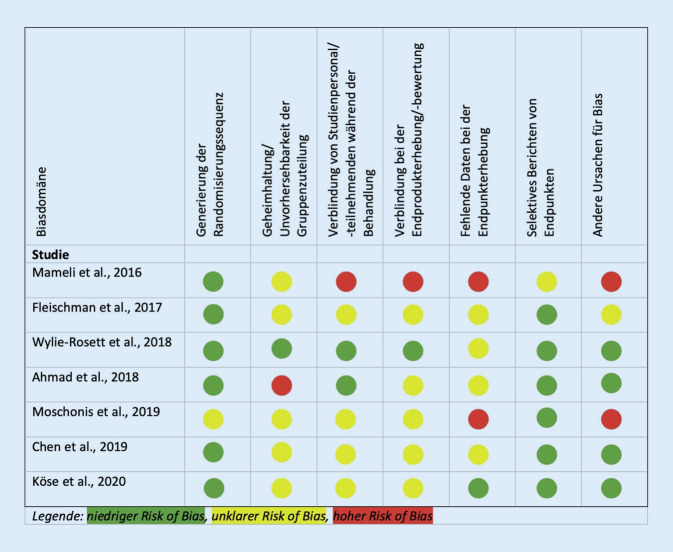

Mit Ausnahme von einer Studie [28] konnte überall eine Randomisierungssequenz generiert werden (Abb. 2). Durch den Studientyp wurden Studienpopulation und -personal entweder nicht [29], einfach [30] oder doppelt [27] verblindet, wobei in 4 Studien keine Angaben hinsichtlich der Vorgehensweise getroffen wurden [25, 26, 28, 31]. Informationen zur Verblindung bei der Endprodukterhebung und -bewertung gab es bei 2 Studien, bei welcher eine kein Risiko [27], die andere ein hohes Risiko für systematische Fehler aufzeigte [29]. Trotz der durchgehend geringen Anzahl an Teilnehmenden wiesen 2 Studien hohe Gefahr für andere Fehlerursachen auf. Hier wiesen eingesetzte Medien fehlende Attraktivität und mangelnde Zielgruppenadaptation der Technologien auf, woraus eine erhöhte Drop-out-Rate resultierte [28, 29].

## Diskussion

Das Ziel der durchgeführten Studie war die Präsentation eines Überblicks über existierende digitale Übergewichts- und Adipositasinterventionen im Kindes- und Jugendalter. Im Rahmen dessen fand eine Analyse eingesetzter Technologien und methodischer Vorgehensweisen statt. Trotz heterogener Mediennutzung bewirkten alle Maßnahmen eine BMI-Verminderung.

### Stärken und Limitationen

Stärken der durchgeführten Analyse liegen in der Einzigartigkeit der Suche, da in der untersuchten Altersgruppe aktuell keine Datenlage über die Effektivität digitaler Adipositasinterventionen besteht. Zudem bildet der Suchzeitraum zwischen 2016 und 2021 neueste Ergebnisse ab. Die Studienlage beinhaltet 3 US-amerikanische, 3 europäische und eine asiatische Studienpopulationen, was die internationale Relevanz der Thematik unterstreicht.

Die Analyse birgt Limitationen. Die Literaturrecherche konnte aufgrund mangelnder abgeschlossener Datenerhebungen lediglich 7 Studien mit erfüllten Inklusionskriterien identifizieren. Präzise Aussagen zur Verteilung verschiedener Übergewichtsgrade der Teilnehmenden innerhalb der Experimental- und Kontrollgruppen wurden nur partiell getroffen, zudem variierte der BMI zu Interventionsbeginn zwischen den Studienpopulationen. Die verschiedenen Interventionsinhalte und -zeiträume sowie die Diversität der Altersverteilung schränken generalisierbare Aussagen über Wirkungsansätze ebenfalls ein. Obwohl sich die Effektivität des Medieneinsatzes abbildet, beeinflussten verschiedene Kulturkreise den geschlechterspezifischen Zugang zu Technologien und das Nutzungsverhalten, was die Replizierbarkeit der Ergebnisse reduziert. Aufgrund der heterogenen Darstellung der BMI-Veränderungen wurde eine einheitliche Schlussfolgerung bezüglich der Interventionseffektivität erschwert.

Die Bewertung des Risikos systematischer Fehler zeigte, dass keine ausreichende Verblindung der Studiendurchführung und der Ergebnisbewertungen durchgeführt wurde. Es ist nicht sichergestellt, ob Messungen und daraus resultierende Schlussfolgerungen davon beeinflusst wurden. Aussagen zur Effektstärke wurden lediglich in einer Studie getroffen [29]. Somit müssen Fehler der potenziellen Wirksamkeitsüberschätzung bestehender Studien in der Ergebnisinterpretation berücksichtigt werden [32]. Die Datenanalyse, -extraktion sowie die Beurteilung des Risikos systematischer Fehler wurden von einer Autorin durchgeführt. Für die Erhöhung der Studienqualität sollten Ergebnisse durch die Sichtung weiterer Autor:innen abgesichert werden.

### Medieneinsatz und Effektivität bei Adipositasinterventionen

Signifikante BMI-Veränderungen wurden in der vorliegenden Analyse beim Einsatz telemedizinischer Maßnahmen [26, 27] sowie SMS für das Smartphone identifiziert [30, 31].

Der Erfolg telemedizinischer Maßnahmen wird in der Literatur partiell belegt. Studienergebnisse von Davis et al. [33] konnten im Kindesalter keinen signifikanten BMI-vermindernden Effekt durch den Einsatz von Telemedizin bestätigen. Zur Begründung wurde darauf hingewiesen, dass für Gewichtsveränderungen längere Interventionsdauern für nachhaltige Ergebnisse vonnöten sind. Zukünftig sollten Frequenz und Länge des Medieneinsatzes genauer untersucht werden, um Rückschlüsse auf eine effektive Dauer der Maßnahmen ziehen zu können. Obwohl die Forderung nach weiteren Studien deutlich wird, bestätigt sich die Praktikabilität und die hohe Akzeptanz telemedizinischer Maßnahmen [34].

Khatami et al. [35] beobachteten bei dem kombinierten Einsatz von einer Website und Erinnerungs-SMS geschlechterspezifische BMI-Veränderungen. Eine höhere Gewichtsreduktion der Mädchen wurde damit begründet, dass die weibliche Studienpopulation ein stärkeres Bewusstsein hinsichtlich des Programmengagements zeigte [36]. Nicht nur sozialkonforme Diskrepanzen, auch Verhaltensunterschiede in der Mediennutzung waren beobachtbar [37]. Aufgrund der höheren Affinität der Mädchen zu analogen Büchern wurde in der Altersgruppe der 11- bis 17-Jährigen eine niedrige Medienfrequenz vermerkt; Smartphones stellten in dieser Gruppe die am häufigsten genutzte Medienquelle dar [38]. Jungen im gleichen Alter zeigten eine hohe tägliche Nutzungsdauer und waren affin gegenüber Spielkonsolen [39].

Studien bestätigten nicht nur eine statische, sondern auch geschlechterspezifische longitudinale Entwicklung der Mediennutzung [40]. Es veränderte sich neben der Medienfrequenzerhöhung auch die Affinität zu präferierten Geräten, da mit steigendem Alter Kompetenzen zur Nutzung weiterer Technologien ermöglicht wurden [41]. Als Konsequenz ist ersichtlich, dass der Einbezug des Geschlechtes und des spezifischen Alters für den Einsatz digitaler Maßnahmen von Interesse ist. Dies spiegelt sich in den Empfehlungen der DAG-Leitlinien für die analogen Behandlungsweisen. Hier wird für effektive Gewichtsveränderungen auf die Berücksichtigung der geschlechterspezifischen Interessen und körperlichen Voraussetzungen hingewiesen [17].

Weitere Studien müssen mediale Maßnahmen auf geschlechts- und altersspezifische Neigungen anpassen und Ergebnisse differenziert betrachten. Um diese komplexen Wirkmechanismen präzise darzustellen, sollten zukünftig zudem neben der Erhebung des BMI auch Veränderungen des (Gesundheits‑)Verhaltens berücksichtigt werden.

Andere Übersichtsarbeiten zur Nutzung digitaler Interventionen bestätigen den positiven Einfluss des Medieneinsatzes auf die BMI-Reduktion, stehen hinsichtlich der eingesetzten Medien jedoch konträr zu vorliegenden Ergebnissen. Mcmullan et al. [20] identifizierten signifikante Einflüsse internetbasierter Maßnahmen und Exergaming, definiert als computergestützte Fitnessspiele zur Förderung der Aktivität. Daraus resultierende signifikante BMI-Veränderungen wurden in weiteren Studien bestätigt [42]. Obwohl der Einfluss von Exergaming durch den hohen Spaßfaktor eine Rücklaufquote von fast 100 % bedingte, gibt es aktuell keine Evidenzlage für 6‑ bis 18-Jährige ohne Vorerkrankungen [43]. Zukunftsweisend empfiehlt sich eine spezifischere Untersuchung dieser Interventionsmöglichkeit.

### Methodische Interventionsgestaltung erfolgreicher Maßnahmen

Während die Nutzung einzelner Medien bereits gewichtsreduzierende Ergebnisse zeigt, weist besonders auch die Kombination digitaler Endgeräte Erfolge vor. Hierbei lässt sich die Mischung von smartphonebasierten Anwendungen und virtuellem Coaching nennen [26, 27, 31]. Auch die Kombination aus digitalem Armband, Erinnerungs-SMS per Smartphone und Onlineschulungen wurde als BMI-reduzierend eingeordnet. Allerdings konnten sich keine signifikanten Veränderungen zeigen [25, 29]. Somit lässt sich das Potenzial der multidimensionalen, medialen Interventionsgestaltung erkennen, jedoch ist eine Analyse spezifischer Kombinationen notwendig.

Fleischman et al. [26] fragten Studienteilnehmende nach ihrer Präferenz hinsichtlich analoger und digitaler Interventionen. 63,6 % der Population bevorzugten virtuelle Beratungen. Trotz der individuellen Zufriedenheit bestätigt die Studienlage die Effektivität der Maßnahmen nicht. In der vorliegenden Studie zeigten Ergebnisse von Mameli et al. [29] in der analogen Kontrollgruppe höhere BMI-Reduktionen als in der digital gestützten Experimentalgruppe. Neben der kleinen Studienpopulation wurde das Fehlen der direkten Face-to-Face-Interaktion als Grund für die Reduktion der Programmeffizienz identifiziert.

Auch Stasinaki et al. [44] postulierten, dass bei kurzzeitigen Gewichtsreduktionsinterventionen eine intensive, persönliche analoge Verhaltensintervention mit Spezialist:innen einen höheren Gewichtsverlust erzielte. In Zukunft ist abhängig von der Interventionsdauer zu überlegen, eine Kombination aus analogen und digitalen Maßnahmen anzubieten zur Wahrung des persönlichen Kontakts zwischen Teilnehmenden und Fachpersonal. Welche Zeiträume welche Interventionsansätze fordern, muss erforscht werden.

Die von Ahmad et al. [30] gestaltete BMI-Reduktionsintervention nutzte soziale Medien über das direkte Umfeld übergewichtiger Kinder. Über Smartphones und Computer wurden Eltern erreicht, da der Zugang der Kinder auf die Facebook- oder WhatsApp-Gruppen durch die Alterslimitierung auf 13 Jahre verwehrt war. Erziehungsberechtigte wurden im Rahmen dessen als Informationenüberträger genutzt, wodurch sie als Multiplikator gesunde Verhaltensweisen weitergaben. Die Effektivität von Familien als Adressaten kommunaler Gesundheitsförderung wurde in der Literatur bestätigt, da eine niedrige Programmpartizipation der Eltern mit einer hohen Drop-out-Rate teilnehmender Kinder korrelierte [29]. Zudem begünstigte die emotionale Bindung und der gemeinsame Lebensstil laut weiterer Studien einen verständlichen Informationstransfer und eine daraus resultierende Gewichtsreduktion [45].

Obwohl die Evidenzlage die Vorteile des familiären Einbezugs stützt, wurde auf den dadurch verstärkten Einfluss des familiären, sozioökonomischen Status hingewiesen [46]. Für Kinder mit schwachem Status verringerte sich die Unabhängigkeit vom prädeterminierten sozialen Umfeld, was die Zugänglichkeit zu Gesundheitsprogrammen reduzierte [47]. Lösungsansätze zur Beseitigung der Stigmatisierungsgefahr wurden in einer niederschwelligen Kommunikation hinsichtlich der Interventionsvorgehensweise festgestellt [45]. In Bezug auf familiär ausgelegte, mediale Interventionen identifizierten Hammersley et al. [48] ein methodisches Problem in der Programmgestaltung, da eine niedrige Verbreitung sozialer Medien unter Erziehungsberechtigten ein Hindernis in der Teilnahme darstellte.

Für kommende Studien ist es somit sinnvoll, die Familien als Gesundheitsmultiplikator zu nutzen. Allerdings sollten Kinder entweder zur Nutzung der Medienkomponente befähigt werden oder Prozesse für Eltern niederschwellig und didaktisch wertvoll aufbereitet werden.

Steigende Programmadhärenz der Teilnehmenden korreliert in der Literatur mit erhöhten Gewichtsveränderungen [49]. In der vorliegenden Studienlage lassen lediglich Ergebnisse von Ahmad et al. [30] Aussagen zum Programmengagement zu, in welchen Adhärenz über die Anzahl von Nachrichten, welche von Eltern gelesen wurden, beschrieben wurde. Andere Studien befragten Teilnehmende nach ihrer Zufriedenheit mit der Intervention oder evaluierten die Anzahl erfolgreich beendeter Onlineprogramme, welche mit einem reduzierten Gewichtsstatus korrelierten [50]. Allerdings fehlt eine Evidenzlage standardisierter Vorgehensweisen der Adhärenzermittlung, welche weitergehend erforscht werden muss.

Neben Ansätzen der methodischen Interventionsgestaltung finden sich Potenziale in der theoretischen Vorgehensweise der Programme. Es ist erkennbar, dass Maßnahmen basierend auf Modellen, wie der sozialkognitiven Theorie, positive Ergebnisse aufweisen [25]. Dies wird im Bereich der analogen Interventionsgestaltung im Rahmen der Adipositasforschung bestätigt. Hierbei wird postuliert, dass verhaltenstherapeutische Techniken (beispielsweise Belohnung oder Verstärkung) das Wissen über Risiken verbessern und den Behandlungserfolg steigern [17]. Im Gegensatz dazu konnte das ADDIE(Analysis, Design, Development, Implementation and Evaluation)-Modell, welches Theorien der Kognition, des Behaviorismus und des Konstruktivismus vereint, nicht zu einer BMI-Reduktion führen [35]. Somit stellt die Konzeption digitaler Interventionen zusätzlich eine Forderung nach der Entwicklung einer wissenschaftlich fundierten Gestaltung.

### Konsequenzen für Forschung und Praxis

Aufgrund steigender Evidenzen zu Konzepten digitaler Übergewichts- und Adipositasinterventionen im Kindes- und Jugendalter ergeben sich Forschungsbereiche, welche zur Weiterentwicklung qualitativ hochwertiger Maßnahmen der Gewichtsreduktion beitragen. Diese sind im Folgenden dargestellt:

1. Durchführung differenzierter Analysen der Subgruppen anhand vorliegender Schlüsselfaktoren wie Interventionstechnologien, Geschlecht, Kulturzugehörigkeit und Altersgruppen,
2. zielgruppenspezifische Adaptation der digitalen Unterstützungsinstrumente zur kindergerechten oder familienfreundlichen Handhabung und leichterem Verständnis,
3. regelmäßige Überprüfung des Erscheinens neuer technologischer Interventionsmaßnahmen, um die in Zukunft erscheinenden Studienergebnisse zeitnah darzustellen.

Die für die Praxis entstehenden Konsequenzen für die Maßnahmengestaltung sind Folgende: Beruhend auf Erkenntnissen bisheriger Studien und analoger Interventionen empfiehlt sich der Einbezug familienbasierter Programme [17]. Zudem erscheint es hilfreich, regelmäßig persönliche Treffen mit adipositasspezifischem Fachpersonal anzubieten, welche digitale Angebote unterstützen. Eine theoretische Fundierung der Programme bietet Vorteile in der kognitiven Zugänglichkeit der Teilnehmenden. Zuletzt weist die momentane Studienlage Lücken hinsichtlich der Kombinationsmöglichkeiten von Medien auf, welche mittels empirischer Überprüfung geschlossen werden müssen.

Während auf individueller Ebene die alltagsnahe Interventionsmöglichkeit die Eigenverantwortung der Kinder hinsichtlich der Programmpartizipation erhöht, verlangt die höhere Mediennutzung eine Ausbildung zusätzlicher digitaler Fähigkeiten [48]. Mit Verbesserung einer solchen Medienkompetenz muss sichergestellt werden, dass Teilnehmende die Maßnahmen hinsichtlich Vorgehensweise und Nutzung verstehen, um das gewichtsreduzierende Interventionsziel medial gestützt wahrnehmen zu können. Hierbei empfiehlt sich die Gestaltung maßnahmengebundener Schulungen, um Anwendungsfehler bei der praktischen Umsetzung digitaler Interventionen zu reduzieren.

Aufgrund der erhöhten Zielgruppenerreichbarkeit stellen digitale Übergewichts- und Adipositasinterventionen auf gesellschaftspolitischer Ebene einen entscheidenden Faktor für die Kindergesundheit dar. Für das Gesundheitssystem resultiert daraus eine Kostenreduktion für Übergewichtsbehandlung und den daraus entstehenden Folgekrankheiten. An- und Abreise für die Betroffenen entfallen, was finanzielle und zeitliche Vorteile birgt. Dank der zu Hause stattfindenden Maßnahmen können Schulungen von Fachpersonal gleichzeitig mehrere Teilnehmende erreichen, wodurch zeitnahe, ressourcensparende Interventionen ermöglicht werden. Trotz der Vorteile liegen in der Kosten-Wirksamkeits-Effektivitätsüberprüfung noch wenige Studiendaten vor. Hier muss in Zukunft eine konkretere Gegenüberstellung durchgeführt werden, welche weiterführend den ethischen Umgang mit den Daten und dem damit verbundenen Datenschutz untersucht.

## Schlussfolgerung

Zusammenfassend wird die Notwendigkeit des Einsatzes digitaler Geräte in der Gestaltung von Adipositas- und Übergewichtsinterventionen deutlich. Neben der erfolgreichen singulären Nutzung von Smartphoneinterventionen bedingen Kombinationen von technischen Instrumenten gesundheitsfördernde Veränderungen. Hier besteht Forschungsbedarf in Bezug auf die effektive Auswahl der Endgeräte. Allerdings unterstreichen vorliegende Studienergebnisse die Relevanz einer analogen Programmbegleitung durch persönliche Treffen mit Fachpersonal, um Verständnis und Partizipation in digitalen Maßnahmen zu steigern. Auch der Einbezug der Familie sowie eine theoretisch fundierte Methodengestaltung bergen Potenziale hinsichtlich einer Gewichtsreduktion. Die geringe Studienpopulation und die teils nicht zielgruppengerechte Anpassung der Maßnahmen auf Alter, Kultur und Geschlecht der Teilnehmenden fordern zukünftig eine gesonderte Gestaltung der medialen Programme. Somit kann die technische Evolution effizient für die gesundheitsfördernde Entwicklung von Kindern und Jugendlichen genutzt werden.
